# Psychosocial Impact of Lockdown on Children due to COVID-19: A Cross-Sectional Study

**DOI:** 10.2174/17450179-v18-e2203210

**Published:** 2022-08-24

**Authors:** Mahdi Alnamnakani, Shuliweeh Alenezi, Hani Temsah, Mohamad Alothman, Rozan Esam Murshid, Hana Alonazy, Haitham Alqurashi

**Affiliations:** 1 Department of Pediatric, King Saud University, King Saud University Medical City, Riyadh, Saudi Arabia; 2 Department of Psychiatry, SABIC Psychological Health Research and Applications Chair (SPHRAC), College of Medicine, King Saud University in Riyadh, Saudi Arabia; 3 Department of Psychiatry, College of Medicine, King Saud University, Riyadh, Saudi Arabia; 4 Department of Pediatrics, King Saud University, King Saud University Medical City, Riyadh, Saudi Arabia; 5 Department of Pediatric Emergency, King Saud University, King Saud University Medical City, Riyadh, Saudi Arabia; 6 Department of Pediatric, King Saud University, King Saud University Medical City, Riyadh, Saudi Arabia

**Keywords:** Evaluation, Impact, Quarantine, Children, COVID-19, Pandemic

## Abstract

**Background:**

Quarantine measures during the COVID-19 lockdown had a negative impact on children’s psychology and development. In this study, we aimed to evaluate the psychological impact of quarantine on children due to the COVID-19 pandemic in Saudi Arabia and to assess types of reported child maltreatment before and after the pandemic.

**Methods:**

A cross-sectional survey among parents was performed along with a retrospective data review for anonymized data from the National Family Safety Program, Saudi Arabia. 436 children participated in this survey during June-November 2020.

**Results:**

The percentage of fathers with an organic or psychological illness in the children with elevated anxiety levels is 18.5% (p-value = 0.019). The anxiety level of the participants was assessed using the Generalized Anxiety Disorder Assessment (GAD-7). Based on the scores, 10.1% had severe anxiety. The depression level of the participants was assessed using Patient Health Questionnaire (PHQ-9). Based on the scores, 4.4% had severe depression. The anxiety level of the children was assessed using Spence Children’s Anxiety Scale – Parent (SCAS-Parent). Based on the overall score, 28.1% of the children had elevated anxiety levels. The anxiety level was elevated in a panic attack and agoraphobia for 36.8% of the kids, in separation anxiety for 26.8%, in physical injury fears for 35.1%, in social phobia for 19%, in obsessive-compulsive for 25.1%, and in generalized anxiety disorder/overanxious for 27.3%.

**Conclusion:**

Quarantine and lockdown during the period of the COVID-19 pandemic have had a negative impact and many adverse effects on the mental and intellectual development of children. These negative outcomes may be addressed *via* well-planned multilevel interventions.

## 1 INTRODUCTION

Severe acute respiratory syndrome (SARS-CoV-2) quickly started to spread globally, causing an international global pandemic that instilled panic and fear among individuals, which resulted in people isolating themselves as well as mandatory quarantine measures. All of these posed a safety risk to children and damaged their mental health, affecting their well-being [1-4].

Children need social interactions in order to have a healthy and stable mental health development. They need to interact with their family, peers, teachers, and friends but these kinds of interactions are not possible during the pandemic where the child is quarantined and isolated; thus, these isolation and quarantine methods may have a bad impact on children’s mental health development [5-13].

Severe acute respiratory syndrome (SARS-CoV-2), also referred to as the novel coronavirus 2019, was discovered in China in the city of Wuhan in December 2019. Patients presenting with pneumonia with varying symptoms were encountered. Some were mild in nature, and some were severely affected.

According to the Saudi Center for Disease Prevention and Control, the worldwide COVID-19 confirmed cases had reached 114,067,979, and as of 1-March-2021, there were 377,383 confirmed cases in Saudi Arabia compared to 4,934 local cases on April 13, 2020 [2]. Thus, many countries, including Saudi Arabia, have implemented many methods in order to counteract this virus, including quarantine on a nationwide basis along with isolation, which was done at home or at a designated governmental facility in order to control the spread of the COVID-19 virus. This method can pose a risk to the general well-being of children [2, 13, 14].

The definition of quarantine that was defined by the centers for disease control and prevention is “Quarantine separates and restricts the movement of people who were exposed to a contagious disease to see if they become sick” [15]. Thus, it prevents the further spread of the virus and ensures safety [13, 14].

During the Ebola virus endemic in West Africa, many young girls were victims of sexual abuse. This can aid our hypothesis that pandemics can have a negative impact on children [4]. Many child abuse cases were reported in Australia, the United States of America, Brazil, and China during the COVID-19 pandemic, which raises concern [4].

A study which was done in India to assess the psychological impact of quarantine on children and adolescents showed that quarantined children were more likely to have psychological issues when compared to those who were not put in quarantine. The study also showed high levels of stress among those who were not quarantined during the COVID-19 pandemic [13].

The SARS-CoV1 epidemic had many psychological and bad mental health outcomes in many children after the epidemic was over, which raises a concern for the COVID-19 pandemic [16-18]. The number of global studies addressing COVID-19 quarantine outcomes on children’s well-being is limited [4, 13]; thus, in this study, we evaluated the psychological impact on children during the COVID-19 pandemic in Saudi Arabia, which will help in issuing protective methods to aid children in need.

## 2 METHODS

A cross-sectional online survey-based study was conducted during June-November 2020. A set of self-rating questionnaires were distributed to parents who live in Saudi Arabia and have children between the ages of 7-18. The data were collected through an online questionnaire that was distributed *via* two online social media applications, WhatsApp and Twitter, using the electronic snowball technique. The sample size was calculated by the calculator.net website and confirmed manually by the following equation n=z2p (1−p)/d2, with a proportion of 50% of parents having no anxiety regarding COVID-19 infection and positive attitude towards school return, z=1.96 (95% confidence level), and (margin of error) d = 5%. The estimated sample size was 385 participants; an additional 20% was added to the original sample size to compensate for non-responsive participants. The final sample size was 436.

### 2.1 Measurements

The survey consisted of 71 questions divided into six sections.

- Sociodemographic characteristics were self-designed and included questions regarding sex, age, place of residence, number of children, region, education level, family’s commitment to the precautionary measures against the COVID-19 virus, and family history of mental illness. Finally, some questions measuring anxiety regarding school return were used.
- The 7-item Generalized Anxiety Disorders Scale Arabic version is developed as a screener for generalized anxiety disorder (GAD). The seven items assessed were: (1) feeling nervous, anxious, or on edge; (2) being able to stop or control worrying; (3) worrying too much about different things; (4) trouble relaxing; (5) being restless; (6) becoming easily annoyed or irritable; and (7) feeling afraid as if something awful might happen. Even though GAD-7 was developed for GAD, it is also used in other anxiety disorders. The GAD-7 is increasingly used as a measure for anxiety in general (Beard and Björgvinsson, 2014) and in anxiety disorder research (Dear *et al*., 2011). It was translated to Arabic and culturally appropriated with good psychometric properties.
- The Patient Health Questionnaire (PHQ) Arabic version is a clinical tool used to screen psychological disorders. Studies have shown that the PHQ is efficient, reliable, and highly acceptable for diagnosing depression, anxiety, and somatic disorders. The Arabic version of the PHQ was found to be valid and reliable for screening depression, anxiety, somatic, and panic disorders in a Saudi sample.
- The Spence Children’s Anxiety parents’ report (Arabic version): The Spence Children’s Anxiety Scale (Spence, 1997) was constructed to evaluate the severity of anxiety symptoms. This measure consists of 44 items, of which 38 reflect specific symptoms of anxiety and six relate to positive filler items to reduce negative response bias. Of the 38 anxiety items, 6 reflect separation anxiety, six social phobias, six obsessive-compulsive problems, six panic, three agoraphobia, six generalized anxiety/overanxious symptoms, and five items concern fears of physical injury. On a 4 point scale from never (0) to always (3), children are asked to rate the frequency with which they experience each symptom.

In this cross-sectional descriptive study, a self-administered questionnaire was used to survey parents across Saudi Arabia in 2020. Inclusion criteria were parents of children (younger than 18 years of age) and living in Saudi Arabia during the COVID-19 pandemic. Exclusion criteria were parents who had no children younger than 18 years at the time of data collection.

Questions were prepared based on the literature referred to earlier. The multidisciplinary team focus group refined the wording and format of the questions before conducting a pilot test with a group of 10 parents for language clarity and consistency.

The survey questionnaire was sent electronically *via* social media groups, and consent to participate was provided on the survey’s first page. Participation in this research was voluntary, and all data were collected confidentially and anonymously.

The questions were clustered into the following four categories:

(1) The first part of the questionnaire consisted of questions regarding the participants’ demographic data, such as age, gender, marital status, education, work, and the number of children.

(2) The second part focused on the family’s health status during COVID-19.

(3) The responding parent completed the GAD7 and PHQ9 scores based on the proceeding 2 weeks.

(4) Finally, the survey system randomly picked one of their children between 6-9 years of age, and the parent was requested to complete Spence Children Anxiety Score for that particular child

Responses were assessed using the Likert scales and frequencies from 1 (totally disagree) to 5 (totally agree).

This study received ethical approval from the Institutional Review Board of King Saud University (#978509287/IRB). Participants were informed that participation was not mandatory and assured of their responses’ anonymity and confidentiality. Consent was obtained from the participants before their enrollment into this survey.

### 2.2 Patient and Public Involvement

The development of the research question and outcome measures were guided by patients’ goals, experiences, and preferences, in which service users influenced the design, content, and face validity of the measure at every stage of development, cooperating with other researchers, clinicians, and stakeholders who were central to this research. We secured a more active patient engagement in the research process to ensure more active patient engagement in the design of this study. Specific steps were advocated to promote patient engagement in 1) defining priorities, 2) study leadership and design, 3) increased access to clinical trials, and 4) information preparation and oversight to participants, 5) dissemination and use of results following post-study review of the patient experience. The findings of research should be distributed in stages rather than waiting for the investigation to be completed. Through emails, we provided research participants with comments on the findings of the study.

### 2.3 Statistical Analysis

Descriptive statistics for the study sample were presented in the form of frequencies and relative frequencies (percent) for categorical variables, while mean and standard deviation were used for representing the numeric variables. Generalized Anxiety Disorder Assessment (GAD-7) was used for estimating the anxiety level of participants, while the Patient Health Questionnaire (PHQ-9) was used for depression. Spence Children’s Anxiety Scale – Parent (SCAS-Parent) was used to assess the anxiety level of the children in 6 domains, and the total score was used to classify the children into elevated anxiety levels and normal anxiety levels based on the 85^th^ percentile. Quantitative variables were expressed by using mean and standard deviation, and categorical variables were expressed by frequency and percentage. Bivariate statistical analysis was carried out using appropriate (*i.e*., student’s t-test, one-way analysis of variance, and Pearson’s correlation) statistical tests based on the type of study and outcome variables. Comparison of the groups was done using the Chi-square test or Fisher’s exact test. IBM SPSS statistics software, version 26, was used for the analysis, and p-value <0.05 was considered statistically significant.

### 2.4 Differences between Children with Special Educational Needs and other Children

For children who have special educational needs, learning problems and/or disabilities, it is more difficult to learn than other participating children of their age. They also have problems with schoolwork, communication, and behavior. The children with SEN were diagnosed with either of the following: speech, language, and communication needs; behavioral, emotional, and social difficulties; autistic spectrum conditions; specific learning difficulties, such as dyslexia and attention deficit hyperactivity disorder (ADHD); moderate learning difficulties; profound and multiple learning difficulties; or multi-sensory impairment.

## 3 RESULTS

After the exclusion of 71 participants that completed the demographic data but not the assessment questions, the total number of participants in this study was 436. The Chi-square test and Fisher exact test were used to compare the characteristics of children with elevated anxiety levels to those without elevated anxiety levels, and the only difference discovered was whether the father was diagnosed with an organic or psychological illness.

**Fig. (1):**
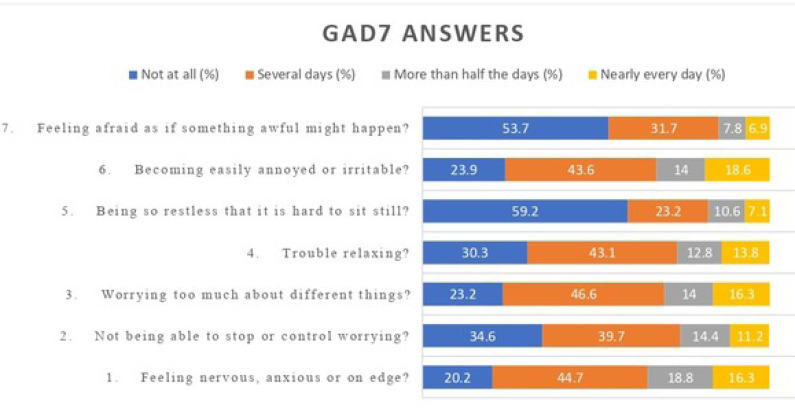
Generalized Anxiety Disorder Assessment (GAD7) answers of participants in percentages.

**Fig. (2):**
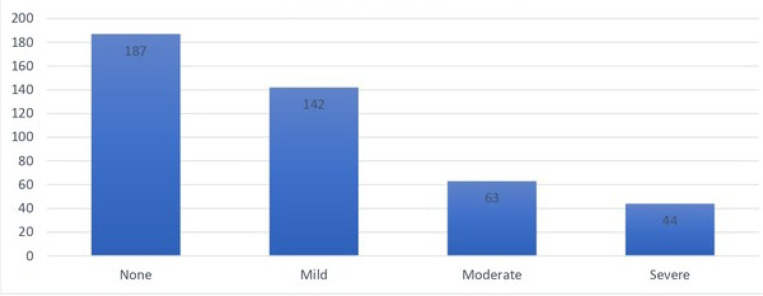
Anxiety level based on GAD-7.

The percentage of fathers with an organic or psychological illness in the children with elevated anxiety levels was 18.5%, which was higher than that in the normal anxiety group, which was 7.8, p-value = 0.019. 66.1% of the participants were mothers, 23.4% were fathers, while 10.6% were other relatives. More than half of the participants (53.7%) said that they were always committed to the precautionary measures against the COVID-19 virus, 35.3% said that they were committed at a medium level, while 2.3% were rarely committed. The majority of the participants were married, *i.e*., 85.3%. Almost half of the participants, 50.5%, were from the age group 35-44. In contrast, 23.9% were from the age group 25-34, 16.5% in the age group 45-54, and lower percentages from upper or lower age groups. The mother and father were alive in most cases (95.4%), and the child lived with both parents in 94.3% of cases. The most common educational level was a university degree, representing 67.2% of mothers and 64.4% of fathers. Saudis represent 92.2% of the study sample, and the monthly income was more than 10000 in 66.3% of the cases. 6.9% of the participants said they lost their job because of COVID-19. 11.2% of the mothers, 10.3% of the fathers, and 8% of the children had an organic or psychological illness. 3.7% of the children were registered in the special needs schooling program, and 25% of the families had persons affected by COVID-19 (Table **1** and Figs. **1**, **2**). Table **2** shows the Pearson correlation.

**Fig. (3):**
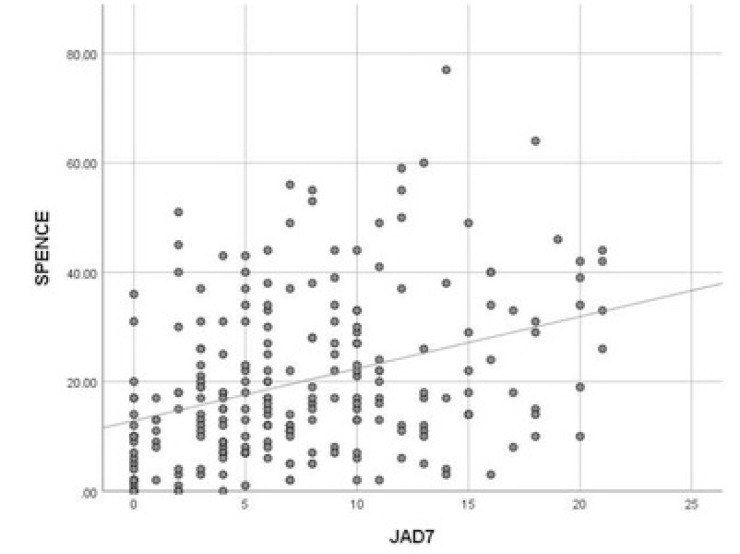
Pearson correlation between child spence score with parents’ GAD7.

**Fig. (4):**
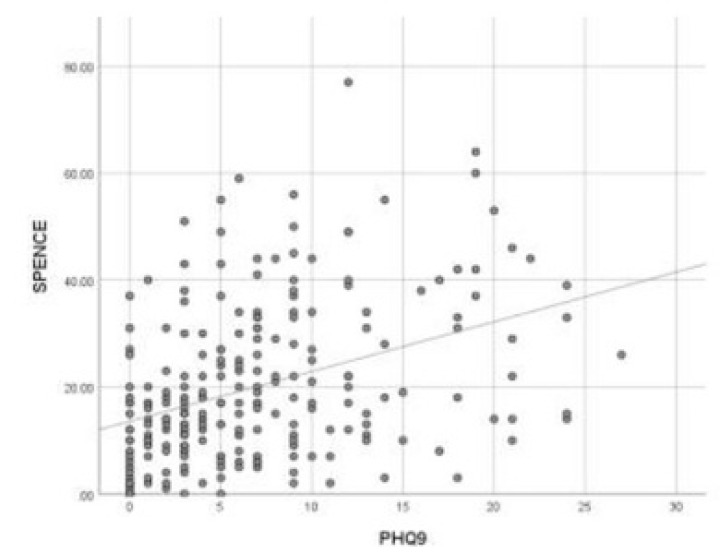
Pearson correlation between child spence score with parents’ PHQ9.

The anxiety level of the participants was assessed using the Generalized Anxiety Disorder Assessment (GAD-7). Based on the scores, 10.1% had severe anxiety, 14.4% had moderate anxiety, 32.6% had mild anxiety, and 42.9% had no anxiety (Table **3** and Figs. **3** and **4**).

Our findings revealed no significant gender differences on the overall mental health score and all its subscale scores, except for aggressive behavior. Boys had a higher mean score on aggressive behavior compared to girls (P-value = 0.002). This means that boys tended to be more aggressive than girls. Also, in terms of age differences, age exerted a significant linear effect and a marginally significant quadratic effect on regulation success, βage = 9.01, t(73) = 2.04, p = .045; β2age β –.23, t(73) = – 1.71, p = .09.

The depression level of the participants was assessed using Patient Health Questionnaire (PHQ-9). Based on the scores, 4.4% had severe depression, 6.2% had moderately severe depression, 14.7% had moderate depression, 32.6% had mild depression, and 42.2% had no depression (Table **4**).

**Fig. (5):**
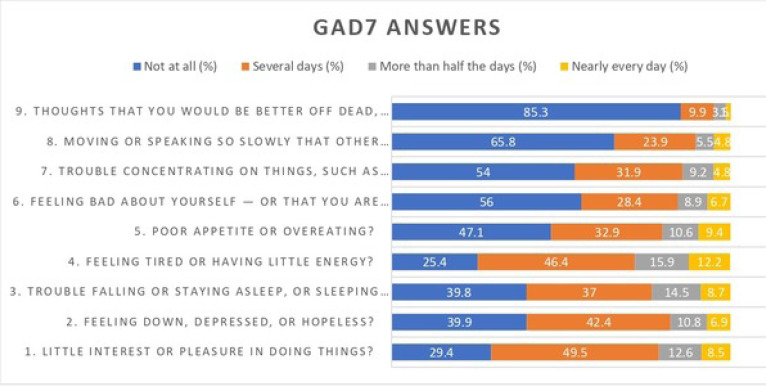
Patient Health Questionnaire (PHQ-9)- GAD 7 answers.

64.9% of the participants in the study had a child whose age was between 6-9 years, and 51.1 of the studied children were girls and 48.9% were boys. The rank of the child in the family was the youngest in 34.6%, the middle child in 36.8%, and the oldest child in 28.6% of cases (Fig. **5**).

**Fig. (6):**
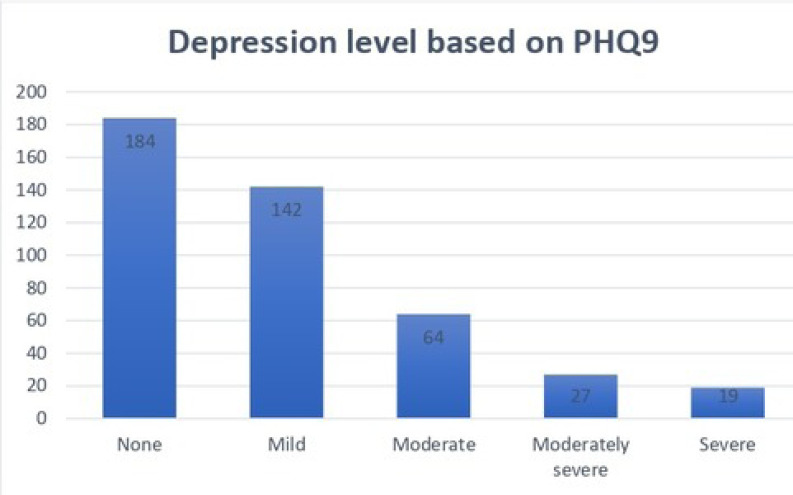
Patient Health Questionnaire (PHQ-9)- GAD 7 answers.

**Fig. (7):**
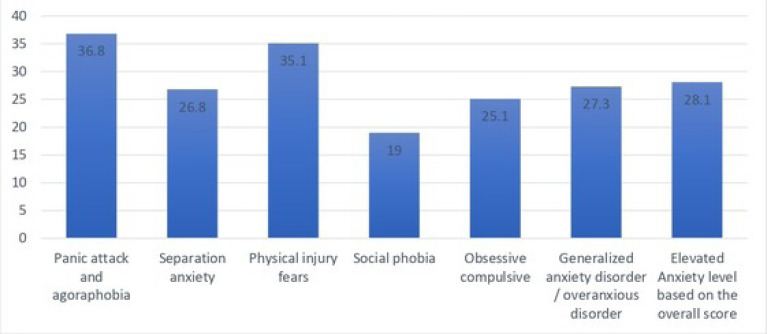
Prevalence of clinically significant types (subscales) of anxiety (%).

The anxiety level of the children was assessed using Spence Children’s Anxiety Scale – Parent (SCAS-Parent). Based on the overall score, 28.1% of the children had elevated anxiety levels. In the six domains, the anxiety level was elevated in a panic attack and agoraphobia for 36.8% of the kids, in separation anxiety for 26.8% of the kids, in physical injury fears for 35.1%, in social phobia for 19%, in obsessive-compulsive for 25.1%, and in generalized anxiety disorder/overanxious for 27.3% of the kids (Figs. **6**, **7**).

Children with elevated anxiety levels were compared to those without elevated anxiety levels using Chi-square and Fisher exact test. The only difference was concerning the father being diagnosed with an organic or a psychological illness (Tables **5** and **6**).

The percentage of fathers with an organic or psychological illness in the children with elevated anxiety level is 18.5%, which is higher than that in the normal anxiety group, which was 7.8, p-value = 0.019 (Tables **7** and **8**).

### 3.1 Correlation between Child Spence Score with Parents’ GAD7 and Parents’ PHQ9

There is a moderate positive correlation between Child SPENCE SCORE with parents’ GAD7 (r=0.35)and parents’ PHQ9 (r=0.38).

Studying the association between anxiety levels in adults based on the JAD7 score and different practices was done using the Chi-square test. The only association was found with feeling anxious regarding sending the child to school after the pandemic. Those with higher anxiety levels tend to be more anxious about sending the child to school.

The association between a child’s anxiety level based on the SPENCE score and different practices was studied using the Chi-square test. There was a statistically significant difference between the child’s academic achievement, the child feeling bored at home, and the family being anxious about sending the child to school. A higher percentage of children with increased anxiety levels have an achievement that is worse than before (58.7%) as compared to children with normal anxiety levels (42.1%). A higher percentage of children with increased anxiety levels have a feeling of being bored at home (82.5%) as compared to children with normal anxiety levels (58.5%). A higher percentage of families of children with increased anxiety levels feel anxious about sending the child to school after the pandemic (55.6%) as compared to families of children with normal anxiety levels (58.5%).

## 4 DISCUSSION

Novel coronavirus 2019 (COVID-19), which is also known as severe acute respiratory syndrome coronavirus 2 (SARS-CoV-2), was first encountered in China in the city of Wuhan in December 2019 as cases of pneumonia, though the presenting symptoms can vary from a mild disease to a severe one. Since then, the novel coronavirus has spread fastly among many countries worldwide, resulting in a pandemic and causing fear among people. Many countries have gone into quarantines and self-isolation, which may pose a risk for child abuse and maltreatment [1, 4]. According to the Ministry of Health of Saudi Arabia, the estimated coronavirus cases were 1,859,011 globally and 4,934 cases locally. As of April 13, 2020, the country has issued quarantine and isolation methods, which may pose a risk for child abuse [2]. According to the WHO, child abuse or maltreatment is defined as “the abuse and neglect that occurs to children under 18 years of age. It includes all types of physical and/or emotional ill-treatment, sexual abuse, neglect, negligence, and commercial or other exploitation, which results in actual or potential harm to the child’s health, survival, development, or dignity in the context of a relationship of responsibility, trust or power” [3].

During the Ebola outbreak in West Africa, there were many reported cases of sexual abuse in young girls. Many countries, including the United States of America, Australia, China, and Brazil, have reported child abuse during the coronavirus pandemic [4]. There are a few studies evaluating the vulnerability of the pediatric population for abuse during the COVID-19 pandemic [4]. Therefore in this study, we evaluated the risks of abuse and the general well-being as well as the state of both the parents and children. Moreover, we evaluated the psychological and social impact of the pandemic as well as the predisposing factors for abuse during the COVID-19 pandemic in Saudi Arabia, which will aid us in developing protective strategies to help those in need.

There are few studies that address the psychological impact of quarantine during the COVID-19 pandemic in children, including Saudi Arabia [13]. The SARS-CoV-1 epidemic that took place between 2002 till 2003 had many reported cases of mental health issues after the epidemic, which raises concerns for the current COVID-19 pandemic regarding possible psychological complications [16-18]. Our study revealed no significant gender differences in the mental health score except on aggressive behavior, which means that boys were more aggressive than girls during the lockdown. The same condition was noticed in age differences, which revealed marginal significance. Our results were similar to another recent study done on 1182 children of different categories (gender, age, education, region, and location), which showed that boys face anxiety, depressive disorders, argument tendency, adverse mental problems, behavioral health symptoms, COVID-19-related trauma, and stressor-related disorder more than girls regardless of their age [19-21].

Evidence shows that violence and vulnerability increase for children during periods of school closures associated with health emergencies. The rate of reported child abuse rises during school closures. Parents and children live with increased stress, media hype, and fear, all of which challenge our capacity for tolerance and long-term thinking. For many, the economic impact of the crisis increases parenting stress, abuse, and violence against children, especially during the quarantine period. Moreover, the separation of the child from his peers and limiting his social interactions pose a risk for many psychological diseases and developmental problems [10, 13].

Violence and maltreatment against children rise during epidemics and pandemics. This might be attributed to many factors such as self-isolation, quarantine, and fear of what is to come. Some people may take this as an opportunity to hurt the victims.

A study was performed in India that addressed the psychological impact of the quarantine measures on children and adolescents from the ages of 9 to 18 and compared them to those who were not quarantined. The study showed that being worried (68.59%) was on top of the list in regards to the feelings that they felt during the quarantine period. Next was feeling helpless (66.11%) and then being afraid (61.98%). The study also showed that those who were quarantined had more mental health problems and more distress in comparison to those who were not quarantined. Nevertheless, the stress levels for those who were not quarantined were also high during the COVID-19 pandemic.

Other feelings experienced by the quarantine group were nervousness, bother, sadness, and anxiety. Many of them also reported sleeping problems. Several of these feelings were attributed to social and financial issues at home, such as a parent who lost their job and was unable to care for the family’s daily living necessities. The feelings of worry also came from the fear that they might infect those around them [13].

Another study, which was done in India, compared the main issues reported with quarantine in adults with those among children. The most common quarantine issue for adults was emotional distress from not being able to leave their homes and not receiving their paychecks because they were unable to go to work, while the main issue with quarantine for children was not being able to go outside for social interactions. The study also compared those who were not quarantined at home and were separated from their family to those who were quarantined at home and suggested that psychological problems might arise more in those who were separated from their family because of fear and anxiety [13].

The study suggested that the use of cell phones and social media can have a positive impact on the quarantine experience because people can communicate with their families and feel more at ease, which, in turn, lowers the psychological impact of the quarantine. The study also recommended that children become more aware of such diseases and measures by watching videos and reading books directed at children on the matter [13].

Another study found that children who were under quarantine were more likely to suffer from mental health disorders such as adjustment disorder, post-traumatic stress disorders, and grief from the loss of one or both parents or another family member. In order to decrease the number of affected children, the Chinese government has issued free-of-charge psychiatric aid by phone, which is accessible throughout the entire day [16].

According to a review published in February 2020, studies showed a negative psychological impact of the quarantine measures, which are a risk factor for the development of many mental health issues, negative feelings and thoughts, distress, post-traumatic stress disorder, anger, and confusion. Several conditions were found to have a negative effect on the individual. The increased levels of stress were the result of fear of being infected, boredom, frustration, and the lack of knowledge about the disease. The longer the duration period of quarantine, the higher was the level of stress.

Being unable to fulfill daily living necessities is a stressor. Moreover, a parent losing their job affects the household financially and psychologically [17]. A qualitative report which was done in Italy to assess the emotional and behavioral aspects of the COVID-19 lockdown in 4 to 10-year-old children showed that 26.48% of the population asked to sleep on their parents’ beds, and 2.84% of the children started to have enuresis.

18.17% of the children describe being afraid, and 5.48% of the population were found to have a decline in their vocabulary. The study also showed that 53.53% were feeling irritable, were intolerant to rules, and had more demands than usual. 21.17% of the children had some mood swings, and 19.99% were suffering from difficulty in sleeping [19].

During the SARS epidemic, children also experienced some forms of maltreatment and abuse. These might be because of parental issues such as stress, anxiety, mental disorders, and substance abuse because parents with these issues were around their children 24 hours a day [20]. A study done in Hong Kong from 2003 to 2004 regarding the SARS pandemic showed that 16 percent of the participants had fear and were feeling helpless. Quarantines might be for a short term period, but the consequences can persist for a long time, especially for the abused children at home. This can include many mental health issues that can arise after the pandemic and problems in behavior [4].

Pandemics and health emergencies, including SARS, swine Flu, and influenza, have been associated with problematic coping behaviors, anxiety, suicide attempts, and mental health disorders, including post-traumatic stress and depressive disorders, with quarantines, social isolation, and limitations on freedom as possible contributing factors [4]. Quarantines can be particularly challenging for parenting, with existing vulnerabilities and abuse magnified for children due to a confluence of school closures, stress, fear, and uncertainty [4].

Violence threatens children’s health and development and can last till adulthood. Traumatic childhood experiences such as violence exposure, abuse, and neglect are common pathways to social, emotional, and cognitive impairment, leading to increased risk of unhealthy behaviors, disability, and premature mortality. These experiences also increase the risk of further victimization and perpetration of violence [13].

For normal psychological development and wellbeing of children, companionship and social interaction are essential components. There is an increased risk of psychiatric disorders whenever there is a separation of children from their caregivers. More importantly, the age of initial separation is known to be relevant to psychological development. It has been realized that quarantine-like measures might have adverse psychological effects on children [13].

Sprang and colleagues reported that children who were isolated or quarantined during pandemics were more likely to develop acute stress disorder, adjustment disorder, and grief. 30% of the children who were isolated or quarantined met the clinical criteria for post-traumatic stress disorder [16].

Epidemiological studies in six developing countries (Colombia, Georgia, Iceland, India, Lebanon, and Russia) showed a prevalence of violence exposure (0.30–0.67), psychological abuse (0.21–0.82), physical abuse (0.13–0.80), sexual abuse (0.03–0.34), and neglect (0.21–0.47). Like findings from other countries, Saudi adolescents are most likely to experience some form of psychological abuse (65%) and least likely to experience sexual abuse (10%), with physical abuse and neglect falling between these and over half reporting that they have experienced neglect or physical abuse [12].

## CONCLUSION

This study aimed to offer an overview of the evidence on mental health and well-being as well as the effects of lockdown on children and young people during both the COVID-19 pandemic and previous comparable events. While research showed mixed results, the conclusions discussed here represent not only evidence on the direct effects of lockdown on the mental health and well-being of children and young people, but also how the problems in the context of families and schooling affect their mental health and well-being. As a consequence, it is proposed that policymakers and those working with children and young people devise and support multidisciplinary and multi-sectoral reactions that alleviate the anxieties and concerns of this group in general while also identifying and supporting those for whom lockdown would have been most difficult.

## Figures and Tables

**Table 1:** Characteristics of the respondents and families.

**Population Characteristics**		**N**	**(%)**
Respondent	Father	102	(23.4)
	Mother	288	(66.1)
	Other relatives	46	(10.6)
How is the family’s commitment to the precautionary measures against the COVID-19 virus	Always committed	234	(53.7)
	Medium commitment	154	(35.3)
	Rarely committed	10	(2.3)
Marital status	Married	372	(85.3)
	Divorced	24	(5.5)
	Widowed	4	(0.9)
	Single (a relative)	36	(8.3)
Age	18-24	16	(3.7)
	25-34	104	(23.9)
	35-44	220	(50.5)
	45-54	71	(16.3)
	55-64	23	(5.3)
	65+	2	(0.5)
Are the mother and father alive?	Yes	416	(95.4)
	No	20	(4.6)
Does the child live with both of his parents?	No	25	(5.7)
	Yes	411	(94.3)
Mother’s education	Primary school	13	(3.0)
	Middle school	13	(3.0)
	Highschool	59	(13.5)
	University	293	(67.2)
	Others	58	(13.3)
Father’s education	Primary school	12	(2.8)
	Middle school	9	(2.1)
	High school	75	(17.2)
	University	281	(64.4)
	Others	59	(13.5)
Mean family’s monthly income	Less than 5000	32	(7.3)
	5000-10000	77	(17.7)
	More than 10000	289	(66.3)
	Prefer not to answer	38	(8.7)
Nationality	Saudi	402	(92.2)
	Non Saudi	34	(7.8)
Job-status	employee	336	(77.1)
	Freelance	41	(9.4)
	Others	59	(13.5)
Have you lost your job because of the COVID-19 pandemic?	No	406	(93.1)
	Yes	30	(6.9)
Has the mother ever been diagnosed with an organic or psychological illness?	No	387	(88.8)
	Yes	49	(11.2)
Has the father ever been diagnosed with an organic or psychological illness?	No	391	(89.7)
	Yes	45	(10.3)
Was the child ever diagnosed with an organic or psychological illness?	No	401	(92.0)
	Yes	35	(8.0)
Was anyone of the family affected by COVID-19?	No	327	(75.0)
	Yes	109	(25.0)
Is the child registered in the special needs schooling program?	No	420	(96.3)
	Yes	16	(3.7)

**Table 2:** Pearson correlation between child spence score with parents’ GAD7 and parents’ PHQ9.

		**JAD7**	**PHQ9**
SPENCE	Pearson Correlation	0.35	0.38
	P-value	<0.001	<0.001
	N	231	231

**Table 3:** Children characteristics

**Characteristics**	-	**N**	**(%)**
Do you have a child whose age is between 6-9 years	No	149	(34.2)
	Yes	283	(64.9)
Rank of the child	The youngest	80	(34.6)
	Middle child	85	(36.8)
	The oldest	66	(28.6)
Child’s age in years	6	57	(24.7)
	7	45	(19.5)
	8	44	(19.0)
	9	85	(36.8)
Gender	Boy	113	(48.9)
	Girl	118	(51.1)

**Table 4:** Spence children’s anxiety scale – parent (SCAS-Parent) - 1

**SCAS items**		**Never (%)**	**Sometimes (%)**	**Often (%)**	**Always (%)**
1	My child worries about things	30.7	45.9	16.9	6.5
2	My child is scared of the dark	34.2	35.9	15.6	14.3
3	When my child has a problem, s(he) complains of having a funny feeling in his/her stomach	61.9	26.8	8.2	3.0
4	My child complains of feeling afraid	38.5	45.0	9.5	6.9
5	My child would feel afraid of being on his/her own at home	29.0	32.5	15.2	23.4
6	My child is scared when s(he) has to take a test	59.7	26.4	8.7	5.2
7	My child is afraid when (s)he has to use public toilets or bathrooms	68.8	18.2	6.1	6.9
8	My child worries about being away from us/me	34.2	36.8	16.5	12.6
9	My child feels afraid that (s)he will make a fool of him/herself in front of people	36.4	36.8	16.0	10.8
10	My child worries that (s)he will do badly at school	36.8	37.7	16.9	8.7
11	My child worries that something awful will happen to someone in our family	55.4	30.7	7.4	6.5
12	My child complains of suddenly feeling as if (s)he can't breathe when there is no reason for this	90.5	7.8	1.3	0.4
13	My child has to keep checking that (s)he has done things right (like the switch is off or the door is locked)	84.8	12.1	2.6	0.4
14	My child is scared if (s)he has to sleep on his/her own	34.6	27.3	18.2	19.9
15	My child has trouble going to school in the mornings because (s)he feels nervous or afraid	67.1	5.2	3.9	5.2
16	My child is scared of dogs	68.0	21.2	5.6	5.2
17	My child can't seem to get bad or silly thoughts out of his/her head	61.0	30.3	6.1	2.6
18	When my child has a problem, s(he)complains of his/her heart beating really fast	70.1	24.7	3.5	1.7
19	My child suddenly starts to tremble or shake when there is no reason for this	93.1	6.1	0.4	0.4
20	My child worries that something bad will happen to him/her	75.8	21.2	3.0	0.0
21	My child is scared of going to the doctor or dentist	54.1	30.3	9.1	6.5
22	When my child has a problem, (s)he feels shaky	70.1	22.1	6.5	1.3
23	My child is scared of heights (e.g., being at the top of a cliff)	60.6	27.3	7.4	4.8
24	My child has to think special thoughts (like numbers or words) to stop bad things from happening	89.6	9.5	0.4	0.4
25	My child feels scared if (s)he has to travel in the car, or on a bus or train	93.5	6.5	0.0	0.0
26	My child worries what other people think of him/her	68.4	25.1	5.2	1.3
27	My child is afraid of being in crowded places (like shopping centres, the movies, buses, busy playgrounds)	77.9	15.6	4.8	1.7
28	All of a sudden, my child feels really scared for no reason at all	84.4	12.6	3.0	0.0
29	My child is scared of insects or spiders	30.7	28.1	20.8	20.3
30	My child complains of suddenly becoming dizzy or faint when there is no reason for this	87.4	11.3	1.3	0.0
31	My child feels afraid when (s)he has to talk in front of the class	64.9	26.0	4.8	4.3
32	My child complains of his/her heart suddenly starting to beat too quickly for no reason	89.2	10.0	0.9	0.0
33	My child worries that (s)he will suddenly get a scared feeling when there is nothing to be afraid of	73.6	22.5	2.6	1.3
34	My child is afraid of being in small closed places, like tunnels or small rooms	69.3	21.2	5.6	3.9
35	My child has to do some things over and over again (like washing his/her hands, cleaning or putting things in a certain order)	82.7	12.1	3.0	2.2
36	My child gets bothered by bad or silly thoughts or pictures in his/her head	66.2	25.5	5.6	2.6
37	My child has to do certain things in just the right way to stop bad things from happening	84.0	10.8	2.6	2.6
38	My child would feel scared if (s)he had to stay away from home overnight	79.7	13.9	3.5	3.0

**Table 5:** Other questions related to COVID-19.

**Questions**		**N**	**(%)**
Has the child visited anyone from his relatives like his grandmother or grandfather during the quarantine period, and how many times did he visit them?	No	76	(34.2)
	Yes, once per month	75	(33.8)
	Yes, once per week	62	(27.9)
	Yes, daily	9	(4.1)
How were the child’s academic achievements during the quarantine period and online studying?	Better than before	25	(11.3)
	The same as before the quarantine	93	(41.9)
	Worse than before	104	(46.8)
The child feels bored at home more than usual	No	77	(34.7)
	Yes	145	(65.3)
Do you feel anxious regarding sending the child to school after the pandemic?	No	140	(63.1)
	Yes	82	(36.9)

**Table 6:** Association between adult’s anxiety levels based on the JAD7 score and different practices.

Association Between Adult’s Anxiety Level Based on The JAD7 Score and Different Practices			Anxiety Level Based on JAD7				P-Value
			None	Mild	Moderate	Severe	
Has the child visited anyone from his relatives like his grandmother or grandfather during the quarantine period, and how many times did he visit them?	No	N	36	20	15	5	0.397
		%	40.9%	27.0%	41.7%	20.8%	
	Yes, once per month	N	27	28	8	12	
		%	30.7%	37.8%	22.2%	50.0%	
	Yes, once per week	N	21	23	12	6	
		%	23.9%	31.1%	33.3%	25.0%	
	Yes, daily	N	4	3	1	1	
		%	4.5%	4.1%	2.8%	4.2%	
How were the child’s academic achievements during the quarantine period and online studying?	Better than before	N	14	8	1	2	0.055
		%	15.9%	10.8%	2.8%	8.3%	
	The same as before the quarantine	N	43	28	16	6	
		%	48.9%	37.8%	44.4%	25.0%	
	Worse than before	N	31	38	19	16	
		%	35.2%	51.4%	52.8%	66.7%	
The child feels bored at home more than usual	No	N	38	24	7	8	0.083
		%	43.2%	32.4%	19.4%	33.3%	
	Yes	N	50	50	29	16	
		%	56.8%	67.6%	80.6%	66.7%	
Do you feel anxious regarding sending the child to school after the pandemic?	No	N	64	46	19	11	0.041
		%	72.7%	62.2%	52.8%	45.8%	
	Yes	N	24	28	17	13	
		%	27.3%	37.8%	47.2%	54.2%	

**Table 7:** Association between child’s anxiety levels based on the SPENCE score and different practices.

Association Between Child’s Anxiety Level Based on the SPENCE Score and Different Practices			Anxiety Level Based on Spence		P-value
			Normal Anxiety Level	Elevated Anxiety Level	
Has the child visited anyone from his relatives like his grandmother or grandfather during the quarantine period, and how many times did he visit them?	No	N	58	18	0.229
		%	36.5%	28.6%	
	Yes, once per month	N	52	23	
		%	32.7%	36.5%	
	Yes, once per week	N	45	17	
		%	28.3%	27.0%	
	Yes, daily	N	4	5	
		%	2.5%	7.9%	
How were the child’s academic achievements during the quarantine period and online studying?	Better than before	N	16	9	0.018
		%	10.1%	14.3%	
	The same as before the quarantine	N	76	17	
		%	47.8%	27.0%	
	Worse than before	N	67	37	
		%	42.1%	58.7%	
The child feels bored at home more than usual	No	N	66	11	0.001
		%	41.5%	17.5%	
	Yes	N	93	52	
		%	58.5%	82.5%	
Do you feel anxious regarding sending the child to school after the pandemic?	No	N	112	28	<0.001
		%	70.4%	44.4%	
	Yes	N	47	35	
		%	29.6%	55.6%	

**Table 8:** Comparison of the characteristics of children with elevated anxiety levels and those without elevated anxiety level.

**Characteristics of Children According To Anxiety Level**		**Anxiety Level**		**P-value**
		**Normal Anxiety Level**	**Elevated Anxiety Level**	
**Family’s commitment to the precautionary measures against the COVID-19 virus**	Always committed	92	34	0.556
		58.2%	55.7%	
	Medium commitment	63	24	
		39.9%	39.3%	
	Rarely committed	3	3	
		1.9%	4.9%	
**Marital status:**	Married	153	57	0.700
		94.4%	91.9%	
	Divorced	7	3	
		4.3%	4.8%	
	Widowed	2	2	
		1.2%	3.2%	
**Are the mother and father alive?**	No	6	5	0.300
		3.6%	7.7%	
	Yes	160	60	
		96.4%	92.3%	
**Does the child live with both of his parents?**	No	8	4	0.744
		4.8%	6.2%	
	Yes	158	61	
		95.2%	93.8%	
**Mean family’s monthly income**	Less than 5000	8	8	0.094
		4.8%	12.3%	
	5000-10000	33	9	
		19.9%	13.8%	
	More than 10000	113	40	
		68.1%	61.5%	
	Prefer not to answer	12	8	
		7.2%	12.3%	
**Has the mother ever been diagnosed with an organic or psychological illness?**	No	147	55	0.417
		88.6%	84.6%	
	Yes	19	10	
		11.4%	15.4%	
**Has the father ever been diagnosed with an organic or psychological illness?**	No	153	53	0.019
		92.2%	81.5%	
	Yes	13	12	
		7.8%	18.5%	
**Was the child ever diagnosed with an organic or psychological illness?**	No	157	59	0.372
		94.6%	90.8%	
	Yes	9	6	
		5.4%	9.2%	
**Was anyone of the family affected by the COVID-19?**	No	122	47	0.855
		73.5%	72.3%	
	Yes	44	18	
		26.5%	27.7%	
**Rank of the child**	The youngest	53	27	0.264
		31.9%	41.5%	
	Middle child	66	19	
		39.8%	29.2%	
	The oldest	47	19	
		28.3%	29.2%	
**Gender of the child**	Boy	86	27	0.160
		51.8%	41.5%	
	Girl	80	38	
		48.2%	58.5%	

## Data Availability

Data is available upon direct request from the corresponding author [M.A].
